# Influenza vaccination for elderly, vulnerable and high-risk subjects: a narrative review and expert opinion

**DOI:** 10.1007/s11739-023-03456-9

**Published:** 2023-10-27

**Authors:** Raffaele Antonelli Incalzi, Agostino Consoli, Pierluigi Lopalco, Stefania Maggi, Giorgio Sesti, Nicola Veronese, Massimo Volpe

**Affiliations:** 1 Gerontology Unit, Department of Internal Medicine and Geriatrics, Campus Bio-Medico University and Teaching Hospital, Rome, Italy; 2 Department of Medicine and Aging Sciences, “G. d’Annunzio” University, Chieti, Italy; 3 Department of Biological and Environmental Sciences and Technologies, University of Salento, Lecce, Italy; 4 Institute of Neuroscience-Aging Branch, National Research Council, Padua, Italy; 5 Department of Clinical and Molecular Medicine, “La Sapienza” University of Rome, Rome, Italy; 6 Department of Internal Medicine, Geriatrics Section, University of Palermo, Palermo, Italy; 7 Department of Clinical and Molecular Medicine, “La Sapienza” University of Rome and IRCCS San Raffaele, Rome, Italy

**Keywords:** Influenza, Burden of disease, Influenza vaccines, Aged, Cardiovascular diseases, Diabetes mellitus, Chronic diseases

## Abstract

Influenza is associated with a substantial health burden, especially in high-risk subjects such as older adults, frail individuals and those with underlying chronic diseases. In this review, we summarized clinical findings regarding the impact of influenza in vulnerable populations, highlighted the benefits of influenza vaccination in preventing severe illness and complications and reviewed the main evidence on the efficacy, effectiveness and safety of the vaccines that are best suited to older adults among those available in Italy. The adverse outcomes associated with influenza infection in elderly and frail subjects and those with underlying chronic diseases are well documented in the literature, as are the benefits of vaccination (mostly in older adults and in patients with cardiovascular diseases, diabetes and chronic lung disease). High-dose and adjuvanted inactivated influenza vaccines were specifically developed to provide enhanced immune responses in older adults, who generally have low responses mainly due to immunosenescence, comorbidities and frailty. These vaccines have been evaluated in clinical studies and systematic reviews by international immunization advisory boards, including the European Centre for Disease Prevention and Control. The high-dose vaccine is the only licensed influenza vaccine to have demonstrated greater efficacy versus a standard-dose vaccine in preventing laboratory-confirmed influenza in a randomized controlled trial. Despite global recommendations, the vaccination coverage in high-risk populations is still suboptimal. All healthcare professionals (including specialists) have an important role in increasing vaccination rates.

**Supplementary Information:**

The online version contains supplementary material available at 10.1007/s11739-023-03456-9.

## Introduction

Influenza infection is a leading cause of morbidity and mortality worldwide, with a substantial health burden. Though typically characterized by pyrexia, myalgia and respiratory tract infection symptoms that generally resolve quickly, seasonal influenza infection may also occur as a severe and life-threatening disease requiring hospitalization, depending on viral- and host-related factors [1]. Morbidity and mortality are greatly increased by secondary bacterial infections or co-infections, and bacterial pneumonia (most commonly caused by infection or co-infection with *Streptococcus pneumoniae*, *Staphylococcus aureus* or *Haemophilus influenzae*) is one of the most common *sequelae* of influenza and main causes of death. The clinical burden of influenza extends beyond pulmonary complications and may involve other organs and systems, resulting in a range of pathological manifestations such as cardiovascular (CV) events, worsening of functional decline and chronic underlying conditions, myositis or rhabdomyolysis and neurological complications [2, 3]. According to data from the Burden of Communicable Diseases in Europe study, influenza was the infectious disease with the highest burden, being responsible for 81.8 median annual disability-adjusted life years (DALYs) per 100,000 population, with subjects aged ≥ 65 years showing the highest group-specific annual burden [4]. Notably, the clinical burden of influenza disproportionately impacts the most vulnerable individuals, such as older adults and subjects with multimorbidity or immunodeficiency of any age. Risk factors associated with increased morbidity and mortality from influenza include age (increased risk of death and hospitalization in subjects aged ≥ 65 years, increased risk of hospitalization in children aged < 5 years), pregnancy, chronic non-communicable disease, immunocompromised state, any medical comorbidity and genetic susceptibility [2]. The Italian Ministry of Health has identified a specific group of subjects as being at high risk for influenza-related complications or hospitalization (see Box in Supplementary Information) [5].

Vaccination is the most effective method for prevention and control of influenza. Evaluation of an influenza vaccine’s benefits for regulatory purposes should include assessment of immunogenicity (although there is no established correlation between immunological parameters and protection against influenza) and efficacy (i.e., the degree to which a vaccine prevents disease, and possibly also transmission, in ideal and controlled circumstances, and therefore measured by randomized controlled trials assessing the reduction in rates of laboratory-confirmed influenza) or effectiveness (i.e., how well the vaccine performs in the real world, therefore mostly observational studies that also assess other end points such as hospitalization and influenza-related pneumonia or mortality, often using a test-negative design). Rigorously conducted studies with a prospective, double-blind, randomized, controlled design are considered the gold standard for assessment of efficacy, and it is important to analyze all available evidence with a standardized and validated methodology. The World Health Organization (WHO) Global Influenza Surveillance and Response System monitors circulating influenza viruses around the world and updates the composition of vaccines twice yearly [6]. Vaccine efficacy/effectiveness is optimal when the vaccine strains match seasonal circulating strains [2]. Older, vulnerable and high-risk individuals exhibit the greatest benefit from vaccination [7–12]. Despite evidence of the benefits of influenza vaccination and global health authorities’ recommendations, vaccination rates remain below target among high-risk subjects. Awareness of the impact of influenza and the benefits of vaccination in high-risk populations appears to be somewhat suboptimal also among healthcare professionals [13], who are seldom actively promoting vaccination.

In this article, we summarized the most relevant findings of studies that examined the risk of adverse outcomes associated with influenza in selected high-risk populations, documenting the benefits of vaccination in averting these outcomes, and reviewed the most recent evidence about efficacy, effectiveness and safety of the vaccines available in Italy that are specifically indicated for the elderly population. The aim of this review is to highlight the benefits of vaccination in high-risk groups and advise clinicians and decision makers about the vaccine types best suited to older adults and high-risk populations, based on evidence from the literature and the opinion of a panel of clinicians with expertise in different areas of medicine.

## Methods

A broad literature search was conducted on PubMed for articles in English language published in the past 10 years pertaining to each of the topics covered in this review. Search terms (all linked with ‘influenza’) included ‘epidemiology’, ‘elderly’, ‘disease burden’, ‘immunosenescence’, ‘frailty’ ‘cardiovascular disease’, ‘diabetes’, ‘pulmonary disease’, ‘respiratory illness’, ‘kidney disease’, ‘liver disease’ ‘vaccination’, ‘vaccine effectiveness’, ‘high-dose vaccines’, ‘standard-dose vaccines’ and ‘enhanced vaccines’. The most relevant articles were then selected and their list of references hand searched for additional publications of interest to be included. The online sites of major international and Italian health authorities (including WHO, US Centers for Disease Control and Prevention [CDC], European Centre for Disease Control and Prevention [ECDC] and Italian Health Ministry) were also checked for up-to-date information pertaining to influenza epidemiology and immunization strategies.

## Impact of influenza on high-risk populations and benefits of vaccination

Among high-risk conditions associated with influenza infection, we focused on older age/frailty, CV diseases and stroke, diabetes mellitus and chronic respiratory, renal and liver diseases. For each condition, we briefly reviewed the biological mechanisms underlying the increased risk of adverse outcomes, as well as the most relevant epidemiological and clinical data regarding the impact of influenza and the benefits of vaccination. Although we reviewed each high-risk group separately for the sake of clarity, multimorbidity is highly prevalent (especially in older adults) and risk factors often overlap. Detailed information about the efficacy, effectiveness and safety of different vaccine types will be provided in the next section.

### Older age and frailty

Elderly subjects are unanimously recognized as being at higher risk for influenza-related complications, hospitalization and death compared with young, healthy adults. It has been estimated that, in recent years, 70–85% of seasonal deaths and 50–70% of hospitalizations associated with influenza have occurred in subjects aged ≥ 65 years, and 309 cases of hospitalization per 100,000 person-years have been reported in this age group [1, 14]. Globally, the estimated mean annual influenza-associated excess respiratory mortality rate ranged from 2.9 to 44.0 per 100,000 persons for subjects aged 65–74 years and from 17.9 to 223.5 per 100,000 for those aged ≥ 75 years, compared with a rate of 0.1–6.4 per 100,000 for subjects aged < 65 years [15]. In a meta-analysis of 234 studies that investigated risk factors for severe or complicated influenza, older age was associated with the highest risk of death during both seasonal and pandemic influenza [16]. In Italy, the Goldstein index-based excess mortality rate, estimated for influenza seasons from 2013 to 2017, was three- to sixfold higher in subjects aged ≥ 65 years compared with the general population, ranging from 65.0 to 147.3 per 100,000 persons in the elderly versus 11.6–41.2 per 100,000 persons in the general population [17]. Among the factors responsible for the increased risk of influenza-related adverse outcomes in the elderly population, a major role is played by frailty (a state of increased vulnerability to stress factors) [18], multimorbidity and immunosenescence. Frailty and chronic diseases are highly prevalent in the elderly population and are associated with an increased incidence of infections and their complications [19]. During recent epidemics in the USA, nine out of ten people hospitalized with influenza had at least one underlying health condition [14]. Age-related immune changes (both in the innate and adaptive immune system), collectively described as immunosenescence and inflammaging, are characterized by disruptions in immune cells activity and biomolecular patterns that induce a less effective immune response to new antigens, such as infective agents and vaccines [19–23]. As reviewed by Vetrano et al. [19], frailty, infections and immunity are interdependent, creating a vicious circle where frailty fosters infections and vice versa, with an altered immune function impairing defense mechanisms. However, reserves in immune function still exist in the elderly population that can be exploited by developing new types of vaccines, such as those containing higher antigen doses or adjuvants [23, 24]. In frail elderly subjects, acute infections such as influenza and secondary bacterial pneumonia affect both physical and cognitive function through multiple mechanisms (i.e., inflammatory response in various organs and systems, prolonged immobilization, decreased nutritional and caloric intake, hypoxia) [3, 19, 25]. In fact, an often underappreciated consequence of influenza in older adults is accelerated functional decline, often leading to loss of independence [3, 25]. Pneumonia has also been associated with an increased risk of dementia [19].

#### Influenza vaccination in older individuals

Literature data regarding the benefits of influenza vaccination in older adults are somewhat controversial, reflecting a variability in vaccine effectiveness that depends on several factors, including virus or vaccine types and host-related factors (i.e., comorbidities and frailty) [14, 26, 27]. In general, there is limited information about protection from hospitalization or mortality in vaccinated versus unvaccinated frail older individuals. Vaccination of elderly subjects with traditional standard-dose vaccines is considered to confer low to moderate protection against influenza and associated adverse outcomes, and there is evidence of a weaker immune response and reduced vaccine effectiveness in older versus younger individuals [23, 28]. The latest Cochrane Review on influenza vaccination in the elderly suggests a vaccine efficacy of 58% against laboratory-confirmed influenza and of 41% against influenza-like illness (ILI) over a single season [26]. Methodological concerns about confounding by indication are often a matter of debate in observational studies of vaccine effectiveness and have been explored in recent investigations [29, 30]. In a register-based study conducted over eight consecutive influenza seasons from 2012 to 2020, confounder-adjusted estimates of vaccine effectiveness against laboratory-confirmed influenza in the elderly population in Finland ranged from 16 to 48% [30]. Several systematic reviews, meta-analyses, case–control and cohort studies have documented a decreased risk of hospitalization and mortality in vaccinated versus unvaccinated subjects aged ≥ 65 years [7, 27, 29, 31, 32]. In Italy, the vaccination coverage of subjects aged ≥ 65 years during the last three seasons was 65.3% (2020–2021), 58.1% (2021–2022) and 56.7% (2022–2023), remaining below the minimum recommended target of 75% [33].

### Cardiovascular diseases and stroke

The association between influenza infection and major CV events is well recognized and has been repeatedly confirmed over the years in epidemiological studies [8, 34, 35]. The link between viral respiratory infections and cardiovascular disease (CVD) is bidirectional. Patients with underlying CVD are at increased risk of cardiopulmonary complications of viral respiratory infections, while viral infections can trigger CV adverse outcomes, mainly by creating a systemic and local inflammatory environment, and promoting secondary infections such as pneumonia, which is in itself associated with increased CV risk through various pathophysiological mechanisms involving the cardiopulmonary system and renal function [8, 34–36]. The most common CV events associated with influenza are acute coronary syndromes and heart failure (HF), but hypertensive crises, cardiogenic shock, acute myocarditis or pericarditis and cardiac tamponade have also been described, though less commonly [37]. Several mechanisms have been proposed in support of the association between viral respiratory infections and acute coronary syndromes, as reviewed by Corrales-Medina et al. [34]. Basically, acute coronary syndromes can be triggered or hastened by the host response to acute infections through generalized inflammatory and thrombogenic changes, as well as local effects on the coronary tree and atherosclerotic lesions [8, 34]. Inflammation plays a central role in triggering acute coronary events. Acute infections can promote prothrombotic conditions, destabilize CVD patients through increased metabolic demands and hypoxia, increase vascular tone via activation of the sympathetic nervous system and induce inadequate coronary artery flow due to fever and tachycardia, thus exacerbating underlying CVD [8, 34]. Proinflammatory cytokines produced during influenza infection are also responsible for an increased risk of HF, mainly by accelerating atherosclerosis, impairing inotropy and inducing adverse cardiac remodeling and an excess production of matrix metalloproteinases tissue inhibitors, which result in ventricular dilatation and increased myocardial collagen content [38].

The increased CV or cerebrovascular risk associated with influenza has been documented in several observational studies (as summarized in Table 1) and systematic reviews. Most of these studies focused on the time-dependent association between a diagnosis of influenza infection (or respiratory tract infection or ILI) and recorded adverse CV outcomes [37, 39–47]. Overall, literature data have demonstrated that there is an association between influenza infection and CVD (mostly acute myocardial infarction and HF), this association is strongest in the first 3 days after exposure to infection, tapering afterwards, and older subjects and those with underlying CVD or other chronic diseases are at increased risk of influenza-related CV deaths or hospitalizations.

**Table 1:** Studies documenting the risk of influenza-related adverse outcomes in cardiovascular diseases, stroke and diabetes

Authors	Study design (period)- country	Subjects included [age]	Main findings
*Cardiovascular diseases/Stroke*			
Meier et al. [36]	Retrospective case–control and case-crossover study (1994–1996)-UK	1922 pts with first-time diagnosis of AMI (no history of clinical factors for AMI), 7649 matched controls [≤ 75 yrs]	Significantly more pts with AMI versus controls had acute RTI in the 10 days before index date (OR 3.6 [95% CI 2.2–5.7] for AMI associated with RTI 1–5 days before index date). In case-crossover analysis, the RR was 2.7 (95% CI 1.6–4.7) for association AMI-RTI in the 10 days before index date
Smeeth et al. [37]	Self-controlled case series (1987–2001)-UK	20,486 subjects with first AMI and 19,063 with first stroke who received influenza vaccine. 20,921 cases exposed to RTI (diagnosed by GP) [median age 72 yrs at first AMI, 78 yrs at first stroke]	The risk of hospitalization for first AMI or stroke was higher after a diagnosis of RTI and was highest during the first three days (IR for days 1–3: AMI 4.95 [95% CI 4.43–5.53]; stroke 3.19 [95% CI 2.81–3.62])
Madjid et al. [38]	Time-series analysis (1993–2000)-Russia	34,892 autopsy-confirmed coronary heart disease deaths (AMI: 11,892, chronic IHD: 23,000) [median age: women 75 yrs, men 65 yrs]	Influenza epidemics were associated with a rise in autopsy-confirmed coronary deaths. In every year, peak AMI and chronic IHD deaths coincided with influenza epidemics and peak acute respiratory disease activity. OR 1.30 (95% CI 1.08–1.56) for AMI and 1.10 (95% CI 0.97–1.26) for chronic IHD in epidemic weeks versus off-season weeks
Warren-Gash et al. [39]	Self-controlled case series (2003–2009)-England/Wales	11,208 pts with first AMI at age ≥ 40 years (3927 with consultation for acute RTI) [≥ 40 yrs; median age 73 yrs]	The risk of hospitalization for AMI was significantly increased during days 1–3 after acute RTI (IR 4.19 [95% CI 3.18–5.53], tapering afterwards. Infections occurring when influenza was circulating or those coded as ILI were associated with higher IRs for AMI (*p* = 0.012)
Nguyen et al. [40]	Time-series analysis (2006–2012, excluding 2009 pandemic season)-USA	88,377 CVD deaths occurring in New York City during nonpandemic influenza seasons (73,363 [83%] in subjects ≥ 65 yrs)	Emergency department visits for ILI were associated with and predictive of CVD mortality. In time-series analyses among subjects ≥ 65 yrs, interquartile range increases in influenza incidence (variously measured) during the previous 21 days corresponded to increases from 2.3% (95% CI 0.7–3.9%) to 6.3% (95% CI 3.7–8.9%) for CVD mortality, and from 2.4% (95% CI 1.1–3.6%) to 6.9% (95% CI 4.0–9.9%) for IHD mortality
Kwong et al. [41]	Self-controlled case series (2009–2014)-Canada	148,307 influenza testing episodes (13% positive), 364 hospitalizations for AMI occurring within 1 year before or after positive testing (control interval). Risk interval: first 7 days after testing [median 77 yrs, IQR 65–86]	There was a significant association between laboratory-confirmed influenza and AMI (IR 6.05 [95% CI 3.86–9.50] for AMI hospitalization during risk interval vs control interval)
Warren-Gash et al. [42]	Self-controlled case series (2004–2014)-Scotland	1227 subjects with first AMI and 762 with stroke who also had laboratory-confirmed RTI within the previous 28 days (risk period) [59–77 yrs]	Rates of AMI and stroke were substantially increased in the week after influenza virus infection (IR 9.80 [95% CI 2.37–40.5] for AMI and IR 7.82 [95% CI 1.07–56.9] for stroke occurring 1–3 days after positive test for influenza versus baseline period
Ohland et al. [43]	Self-controlled case series (2010–2016)-Denmark	606 subjects with first AMI and 744 with stroke who also had laboratory-confirmed RTI within the previous 28 days (risk period) [AMI: 65% of subjects aged ≥ 65 yrs; stroke: 71% aged ≥ 65 yrs)	There was a significant CV event triggering effect following RTI (IR 17.5 [95% CI 8.5–36.2] for AMI and 10.3 [95% CI 4.2–25.4] for stroke occurring 1–3 days after positive test for influenza; IR 5.1 [95% CI 1.6–16.3] for AMI and 6.5 [95% CI 2.4–17.7] for stroke occurring 4–7 days after test versus baseline period)
Chow et al. [34]	Cross-sectional analysis of data from FluSurv-NET (2010–2018 influenza seasons)-USA	80,261 hospitalized adults with laboratory-confirmed influenza [median age 69 yrs, interquartile range 54–81 yrs]	11.7% of hospitalized pts had an acute CV event, mostly HF (6.2%) or IHD (5.7%). Older age and underlying CVD were associated with higher risk for acute CV events. Among pts with chronic CVD, 20.6% had an acute CV event
Moa et al. [44]	Epidemiological study based on generalized-additive statistical model (2001–2018)-Australia	Subjects hospitalized for sudden cardiac arrest (SCA): 30,822 among those aged 50–64 yrs and 91,205 among those aged ≥ 65 yrs	A significant association was found between SCA hospitalizations and laboratory-confirmed influenza notifications in some years in older subjects. Estimated average annual SCA hospitalization rate attributable to influenza per 100,000 population was 5.3 (95% CI 4.4–6.2) in subjects aged ≥ 65 yrs
*Diabetes*			
Allard et al. [45]	Retrospective study (25May–1Jul 2009)-Canada	239 hospitalized pts with PCR- confirmed A(H1N1) influenza	DM tripled risk of hospitalization (prevalence ratio 3.10 [95% CI 2.04–4.71]) and quadrupled risk of ICU admission (OR 4.29 [95% CI 1.29–14.3]) versus subjects without DM
Campbell et al. [46]	Retrospective study (Apr–Sep 2009)-Canada	1479 hospitalized pts with laboratory-confirmed A(H1N1) influenza	The risk of a severe outcome (ICU admission or death) was greatest for pts with DM (RR 2.2 [95% CI 1.7–2.7])
Wilking et al. [47]	Retrospective study (2009–2010)-Germany	252 fatal cases with laboratory-confirmed A(H1N1) influenza [median 47 yrs, IQR 29–57]	DM doubled mortality risk versus subjects without DM (RR 2.3 [95% CI 95% 1.5–3.6])
Lenzi et al. [48]	Retrospective study (2010)-Brazil	4740 pts with laboratory-confirmed A(H1N1) influenza	DM was identified as a risk factor for hospitalization (OR 3.04 [95% CI 2.03–4.55]; *p* < 0.001)
Lau et al. [49]	Population-based cohort study (2000–2008)-Canada	56,513 working-age subjects with DM [median 51 yrs] versus 110,202 nondiabetic controls [median 50 yrs]	Subjects with DM had a 6% higher risk for all-cause hospitalization associated with influenza (RR 1.06 [95% CI 1.02–1.10]) versus nondiabetic subjects
Ruiz et al. [50]	Nationwide population-based cohort study (2009–2013)-Norway	149,432 subjects aged > 30 yrs with T2DM [mean 65.2 yrs] versus 2,842,796 subjects without T2DM [mean 53.2 yrs]	T2DM doubled the risk of hospitalization for pandemic influenza (HR 2.46 [95% CI 2.04–2.98]) versus subjects without T2DM. The relative increase in mortality associated with hospitalization was lower in subjects with T2DM (HR 1.82 [95% CI 1.21–2.74]) than in those without T2DM (HR 3.89 [95% CI 3.27–4.62])
Owusu et al. [51]	Cross-sectional analysis of data from FluSurv-NET (2012–2017 influenza seasons)-USA	31,934 hospitalized adults aged ≥ 65 yrs with laboratory-confirmed influenza [median age 80 yrs, IQR 72–87]	34% of all hospitalized pts had DM. Compared with pts without DM, those with DM had higher rates of influenza-associated hospitalization (RR 1.57, 95% CI 1.43–1.72), ICU admission (RR 1.84, 95% CI 1.67–2.04), pneumonia (RR 1.57, 95% CI 1.42–1.73) and in-hospital death (RR 1.48, 95% CI 1.23–1.80)

*AMI* acute myocardial infarction; *CI* confidence interval; *CV* cardiovascular; *CVD* cardiovascular disease; *DM* diabetes mellitus; *GP* general practitioner; *HF* heart failure; *HR* hazard ratio; *ICU* intensive care unit; *IHD* ischemic heart disease; *ILI* influenza-like illness; *IQR* interquartile range; *IR* incidence ratio; *OR* odds ratio; *RR* relative risk; *RTI* respiratory tract infection; *SCA* sudden cardiac arrest; *T2DM* type 2 diabetes mellitus

#### Influenza vaccination in CVD

There is a large body of evidence, from population-based observational studies, randomized controlled trials (RCTs; summarized in Table 2) and systematic reviews, attesting to the protective effects of influenza vaccination in subjects with CVD [9, 37, 52–60]. Robust evidence of the CV protective effects of vaccination is provided by a large RCT [55], which was included in a recent meta-analysis of six RCTs published between 2000 and 2021, comprising 9001 patients who received influenza vaccination or placebo/control [9]. Overall, vaccination was associated with a 34% lower risk of major adverse CV events (MACEs), corresponding to a number needed to vaccinate of 56 patients to prevent a MACE. Higher-risk patients with a recent acute coronary syndrome benefited most from vaccination, with a 45% lower risk of MACEs. A few studies have explored the association between influenza vaccination and CV outcomes specifically in the HF population [56, 60–62]. In a sub-analysis of the Prospective Comparison of ARNI with ACEI to Determine Impact on Global Mortality and Morbidity in Heart Failure (PARADIGM-HF) trial, vaccination against influenza was associated with a reduced risk for all-cause mortality compared with unvaccinated subjects in propensity adjusted models (hazard ratio 0.81, 95% CI 0.67–0.97; *p* = 0.015), while statistical significance was not reached for the composite outcome of CV death and HF hospitalization [38, 61]. Influenza vaccination was associated with a 18% reduced risk of both all-cause mortality and CV mortality in a nationwide cohort study from Denmark which included all registered HF patients (*n* = 151,328) [62]. A large pragmatic RCT in HF subjects found no significant effect of influenza vaccination versus placebo relative to the primary CV and cerebrovascular outcomes, but suggested a clinical benefit of vaccination in terms of reduced incidence of pneumonia and hospitalizations, as well as reduced CV events and mortality during peak influenza periods [57]. In a RCT that compared the effectiveness of a trivalent HD-IIV versus a quadrivalent SD-IIV in a high-risk population with pre-existing CVD, no significant differences emerged between the two strategies in reducing all-cause mortality or cardiopulmonary hospitalization [56]. Several potential factors may have contributed to these findings, apart from the broader strain coverage conferred by the quadrivalent vaccine [56].

**Table 2:** Main randomized controlled trials investigating the benefits of influenza vaccination in cardiovascular diseases (statistically significant relative risk/hazard ratios in gray)

Author study name-country (period)	Participants (mean age-% women)	Intervention (blinding)	Outcomes (follow-up)	Main findings
Gurfinkel et al. [52] FLUVACS-Argentina (2001)	301 inpatients with recent MI or stable CAD and planned PCI (65 yrs-31%)	Trivalent IIV versus no vaccination	PE: CV death SE: composite of CV death, MI and rehospitalization for severe recurrent ischemia (12 months)	PE: RR 0.34 (95% CI 0.17–0.71; *p* = 0.002) SE: RR 0.59 (95% CI 0.4–0.86; *p* = 0.004)
Ciszewski et al. [53] FLUCAD-Poland (2004–2005)	658 outpatients with angiography-confirmed CAD (60 yrs-27%)	Trivalent SD-IIV versus no vaccination (double-blind)	PE: CV death SE: 1) MACE [composite of CV death, acute MI and coronary revascularization]; 2) coronary ischemic event [MACE or hospitalization for myocardial ischemia (12 months)	PE: HR 1.06 (95% CI 0.15–7.56; *p* = 0.95) SE: 1) HR 0.54 (95% CI 0.24–1.21; *p* = 0.13); 2) HR 0.54 (95% CI 0.29–0.99; *p* = 0.047)
Phrommintikul et al. [54] Thailand (2007–2008)	439 inpatient with ACS within 8 weeks (66 yrs-44%)	Trivalent IIV versus no vaccination (open with blinded endpoint)	PE: MACE [composite of death, hospitalization for ACS, HF and stroke] SE: CV death (12 months)	PE: HR 0.70 (95% CI 0.57–0.86; *p* = 0.004) SE: 1) HR 0.39 (95% CI 0.14–1.12; *p* = 0.088)
Fröbert et al. [55] IAMI-Sweden, Denmark, Norway, Latvia, UK, Czech Republic, Bangladesh, Australia (2016–2020)	2532 patients with recent MI and completed coronary angiography or PCI, or high-risk stable CAD (59.9 yrs-18%)	Trivalent or quadrivalent SD-IIV versus placebo (double-blind)	PE: composite of all-cause death, MI or stent thrombosis SE: hierarchical testing for all-cause death, CV death, MI and stent thrombosis (12 months)	PE: HR 0.72 (95% CI 0.52–0.99; *p* = 0.040) SE: all-cause death HR 0.59 (95% CI 0.39–0.89; *p* = 0.010), CV death HR 0.59 (95% CI 0.39–0.90; *p* = 0.014), MI HR 0.86 (95% CI 0.50–1.46; *p* = 0.57), stent thrombosis HR 1.94 (95% CI 0.48–7.76; *p* = 0.34)
Vardeny et al. [INVESTED] [56] USA–Canada (2016–2019)	5260 patients with recent MI or HF hospitalization and ≥ 1 additional risk factor (mean 65.5 yrs-28%)	Trivalent HD-IIV versus quadrivalent SD-IIV	PE: composite of all-cause death, or hospitalization for CV or pulmonary causes during each season SE: 1) CV death, or hospitalization (each season); 2) all-cause death; 3) cardiopulmonary hospitalization and all-cause death (all seasons); 4) first cardiopulmonary hospitalization and all-cause death (all seasons)	PE: HR 1.06 (95% CI 0.97–1.17; *p* = 0.21); SE: 1) HR 1.08 (95% CI 0.97–1.20; *p* = 0.16); 2) HR 1.01 (95% CI 0.84–1.21; *p* = 0.96); 3) HR 1.04 (95% CI 0.94–1.15; *p* = 0.44); 4) HR 1.05 (95% CI 0.96–1.15; *p* = 0.26)
Loeb et al. [57] IVVE-10 countries in Asia, the Middle East and Africa (2015–2021)	5129 patients with NYHA class II-IV HF (mean 57.2 yrs-51.4%)	Trivalent* SD-IIV versus placebo (double-blind)	PE: 1) first-event composite for CV death, non-fatal MI and non-fatal stroke; 2) recurrent-events composite for CV death, non-fatal MI, non-fatal stroke and hospitalization for HF failure SE: all-cause death, CV death, non-fatal MI, non-fatal stroke, all-cause hospitalization, hospitalization for HF failure and pneumonia (3 years)	PE: 1) HR 0.93 (95% CI 0.81–1.07; *p* = 0.30); 2) HR 0.92 (95% CI 0.84–1.02; *p* = 0.12) SE: all-cause hospitalization HR 0.84 (95% CI 0.74–0.97; *p* = 0.013), pneumonia HR 0.58 (95% CI 0.42–0.80; *p* = 0.0006) Peak circulating influenza periods analysis: PE: 1) HR 0.82 (95% CI 0.68–0.99; *p* = 0.038) 2) HR 0.88 (95% CI 0.74–1.03; *p* = 0.11) SE: all-cause death HR 0.79 (95% CI 0.66–0.95; = 0.0099), CV death HR 0.77 (95% CI 0.63–0.94; 0.0099), pneumonia HR 0.51 (95% CI 0.32–0.81; *p* = 0.0034)

*2.3% of vaccines administered were quadrivalent SD-IIV

*ACS* acute coronary syndrome; *CAD* coronary artery disease; *CI* confidence intervals; *CV* cardiovascular; *FLUCAD* influenza vaccination in prevention from acute coronary events in coronary artery disease; *FLUVACS* flu vaccination acute coronary syndromes; *IAMI* influenza vaccination after myocardial infarction; *HD* high dose; *HF* heart failure; *HR* hazard ratio for vaccine versus control/placebo; *IIV* inactivated influenza vaccine; *INVESTED* influenza vaccine to effectively stop cardiothoracic events and decompensated heart failure; *IVVE* influenza vaccine to prevent adverse vascular events; *MACE* major cardiovascular adverse events; *MI* myocardial infarction; *NYHA* New York Heart Association; *PCI* percutaneous coronary intervention; *PE* primary end point; *RR* relative risk for vaccine versus control; *SD* standard dose; *SE* secondary end point(s)

Taken together, literature data indicate that the reduction in the risk of CV adverse events associated with influenza vaccination is comparable with—or even greater than—that achievable with established CV therapies such as aspirin, angiotensin-converting enzyme inhibitors, β-blockers, statins or anti-platelet therapy [9]. Therefore, influenza vaccination should be included as a first-line intervention among CV prevention strategies, as recommended in the guidelines of international and national cardiologic scientific societies [8, 63].

### Diabetes

Subjects with diabetes are a well-known high-risk group for influenza-related adverse outcomes, for whom vaccination against influenza is recommended by the WHO and all major public health authorities [64, 65]. There is evidence of an association between diabetes and infectious diseases, with a higher incidence and a more severe course of infections in diabetic patients [64, 66]. Diabetes was found to be a risk factor for premature death caused by multiple infections, is a frequent underlying disease in patients with community-acquired pneumonia and is also considered a risk factor for severe bacteremia upon infection with *S. pneumoniae* or in the course of pneumonia caused by other pathogens [65, 67]. Although the pathophysiologic conditions underlying the increased severity of influenza associated with diabetes are not fully understood, there is a growing body of evidence that hyperglycemia and glycemic oscillations can increase the severity of bacterial and viral infections through several mechanisms that may include immunosuppressive effects, elevated airway glucose concentrations, reduced pulmonary function, enhanced cytokine production and overexpression of adhesion molecules in pulmonary endothelial cells [66, 68]. Comorbidities that are highly prevalent in people with diabetes and include CVD, chronic kidney disease and obesity are another important contributor to the increased severity of influenza infection [64, 68]. Influenza-related adverse outcomes observed in diabetic patients are often driven by the impact of viral infections on the CV system [37, 44].

The largest body of evidence regarding enhanced influenza severity in subjects with diabetes was published in the aftermath of the 2009–2010 A(H1N1) pandemic, although the relationship between diabetes and severe influenza had also emerged previously [68]. This relationship, however, has not always been evident, especially during seasonal influenza of subtypes other than A(H1N1), such as A(H3N2) [65]. The findings of numerous observational studies from several countries that have documented a significantly increased risk of influenza-related adverse outcomes in diabetic versus nondiabetic subjects are summarized in Table 1 [48–51, 69–71]. Notably, the increased risk for influenza-related adverse outcomes has been documented not only in elderly persons, but also in younger diabetic subjects, who are not usually a target of vaccination programs [69]. Overall, data from observational studies show evidence that diabetes increases the risk of influenza-related hospitalization and death (although not consistently across different seasons), *sequelae* and complications of severe influenza associated with diabetes are more common in elderly people, but can also affect younger, working-age subjects, and comorbidities such as obesity, CVD and chronic kidney disease are highly prevalent among diabetic hospitalized subjects.

#### Influenza vaccination in diabetes

Annual influenza vaccination of all subjects with diabetes should be an integral part of preventive health strategies, as currently recommended [5]. Although the immune responsiveness of diabetic subjects to vaccination has been questioned, most of the immunogenicity studies have demonstrated that this population too can achieve effective and sustained humoral and cellular immune responses, similar to those observed in nondiabetic subjects [64, 72, 73]. Currently available data on the protective effects of influenza vaccination in diabetic subjects support the benefits of annual vaccination in this population, despite the methodological limitations of many studies and the difficulties in quantifying the extent of protection provided by vaccination due to variable levels of circulating viral strains in different seasons and the risk of vaccine mismatch [65, 72, 74, 75]. The reported benefits of vaccination in the diabetic population consist mostly in reduced rates of hospitalization or mortality versus unvaccinated subjects (Table 3) [10, 70, 76–79]. The reduction of the risk of adverse outcomes in vaccinated subjects was also observed in younger diabetic subjects [77, 78], and was found to extend to specific CV outcomes [10, 79]. In a meta-analysis of four cohort studies and two case–control studies, influenza vaccination in adult and elderly patients with diabetes was associated with a lower mortality rate (Mantel–Haenszel odds ratio [MH-OR] 0.54, 95% CI 0.40–0.74; *p* < 0.001) and a lower risk of hospitalization for pneumonia (MH-OR 0.89, 95% CI 0.80–0.98; *p* = 0.18) [11]. Influenza vaccination coverage in the diabetic population is still below the minimum recommended range of 75% in Italy, especially for subjects aged 18–64 years (32.7% in 2020–2021) [80].

**Table 3:** Studies investigating the benefits of influenza vaccination in diabetes

Authors country (period)	Study design	Participants [age-% female]	Intervention	Main findings
Heymann et al. [76] Israel (2000–2001; winter and summer periods)	Observational outcome-research study (data from health maintenance organization)	15,556 pts with DM aged ≥ 65 yrs (mean 73 yrs-48% among vaccinated subjects) and 69,097 subjects without chronic diseases [mean 75 yrs-53% among vaccinated subjects] as reference group	Influenza vaccine. Vaccination coverage: 48.8% among DM pts, 42.0% among reference group	Vaccination was associated with a reduction of hospitalization rates by 12.3% in DM pts and 23.0% in nondiabetic subjects (*p* = 0.08). Among DM pts, there was significantly reduced mortality for vaccinated versus unvaccinated pts (OR 0.35 [95% CI 0.25–0.49; *p* < 0.001 for men and OR 0.32 [95% CI 0.20–0.50; *p* < 0.001]) for women
Looijmans-Van den Akker et al. [77] The Netherlands (1999–2000)	Nested case–control study, part of the primary care-based PRISMA study	Among 9238 pts with DM, 192 cases died (*n* = 61) or were hospitalized (*n* = 131), and were compared with 1561 control subjects [≥ 18 yrs-48% among cases, 62% among control subjects]	Trivalent IIV. Vaccination coverage: 73.4% among cases, 85.8% among control group	Vaccination was associated with a reduction in hospitalization by 54% (95% CI 26–71%; *p* = 0.002) and mortality by 58% (95% CI 13–80%; *p* = 0.019). Reductions in hospitalization or death were higher in subjects aged 18–64 yrs (72%) than in those aged ≥ 65 yrs (39%). No significant difference was observed between first-time or repeat vaccination
Lau et al. [78] Canada (2000–2008)	Population-based cohort study (administrative data from Manitoba)	91,605 adults with DM, of whom 56,513 were of working age (< 65 yrs) and a control group without DM, for a total of 543,367 person-years follow-up Comparisons between working-age DM [median 53 yrs, 48%], elderly DM [median 74 yrs, 53%] and control subjects [median 74 yrs, 56%]	Influenza vaccine. Vaccination coverage: 35% among working-age DM, 54% among elderly DM and 48% among control subjects	In working-age DM subjects, vaccination was associated with relative reductions in pneumonia/influenza hospitalizations by 43% (95% CI 28–54%; *p* < 0.001) and all-cause hospitalizations by 28% (95% CI 24–32%; *p* < 0.001) but not ILI. Similar VE for pneumonia/influenza hospitalizations (45–55% reductions) and all-cause hospitalizations (33–34% reductions) observed in the elderly population, regardless of DM status
Vamos et al. [79] England (2003–2010)	Retrospective cohort study (primary- and secondary-care data from the Clinical Practice Research Datalink in England	124,503 adults with T2DM (7 annual cohorts), contributing 623,591 person-years [mean 56–66 yrs, 45–49% in 2 seasons]	Influenza vaccine. Vaccination coverage: 63.1–69.0%	During influenza seasons, vaccination was associated with significantly lower rates of hospitalization for stroke (IRR 0.70 [95% CI 0.53–0.91]), HF (IRR 0.78 [95% CI 0.65–0.92]) and pneumonia/influenza (IRR 0.85 [95% CI 0.74–0.99]), and significantly reduced rates of all-cause death (IRR 0.76 [95% CI 0.65–0.83]) versus non-vaccinated subjects. The change in hospitalization rates for acute MI was not significant
Ruiz et al. [70] Norway (2009–2013)	Nationwide population-based, cohort study	149,432 subjects aged > 30 yrs with T2DM [mean 65.2 yrs] versus 2,842,796 subjects without T2DM [mean 53.2 yrs]	AS03-adjuvanted influenza vaccine with A/California/07/2009 (H1N1) strain. Vaccination coverage (pandemic influenza): 59.4% among T2DM subjects	Compared with non-vaccinated subjects with T2DM, vaccinated subjects had lower rates of hospitalization (HR 0.22 [95% CI 0.11–0.39]) and mortality (HR 0.75 [95% CI 0.73–0.77]) for pandemic influenza. Corresponding estimates for vaccinated versus non-vaccinated subjects without T2DM were HR 0.41 (95% CI 0.33–0.52) for hospitalization and HR 0.91 (95% CI 0.90–0.92) for mortality
Modin et al. [10] Denmark 2007–2016)	Nationwide population-based, cohort study	241,551 pts with DM and without IHD, HF, COPD, cancer or previous cerebrovascular disease (425,318 person-years follow-up) were monitored for a median of 4 influenza seasons [mean 58.7 yrs-47.1%]	Influenza vaccine. Vaccination coverage: 24–36%	Vaccination was associated with significantly reduced risks for all-cause death (HR 0.83 [95% CI 0.78–0.88; *p* < 0.001]), CV death (HR 0.84 [95% CI 0.77–0.91; *p* < 0.001]), death due to stroke or acute MI (HR 0.85 [95% CI 0.74–0.98; *p* = 0.028]) and hospitalization due to DM-related acute complications (HR 0.89 [95% CI 0.83–0.97; *p* = 0.006])

*CI* confidence interval; *COPD* chronic obstructive pulmonary disease; *DM* diabetes mellitus; *IIV* inactivated influenza vaccine; *HF* heart failure; *HR* hazard ratio; *IHD* ischemic heart disease; *IRR* incidence rate ratio; *MI* myocardial infarction; *OR* odds ratio; *PRISMA* Prevention of Influenza; Surveillance and Management; *T2DM* type 2 diabetes mellitus; *VE* vaccine effectiveness

### Chronic respiratory diseases

People with underlying chronic respiratory diseases, such as asthma, chronic obstructive pulmonary disease (COPD) and chronic bronchitis, are a high-risk group for complicated or severe influenza. According to US CDC data, 32.5% of adults hospitalized with influenza-related conditions had chronic lung disease during the 2021–2022 season [14]. Asthma is the most common underlying condition in children hospitalized with influenza and is highly prevalent in adults too, with reported rates of 7.6–46% in adults for influenza-related admission to healthcare facilities [14, 81]. Influenza infection can increase inflammation of the airways, thus worsening symptoms, and trigger asthma attacks [14]. Although asthma patients were generally reported to be at higher risk for hospitalization or intensive care unit (ICU) admission during influenza seasons, a number of clinical studies found that subjects with asthma had a less severe disease compared with non-asthmatic patients [3]. Pre-admission corticosteroid treatment, pulmonary immune responses and a lower threshold for hospitalization in asthma patients might partly explain (among other hypotheses) these unexpected findings [3, 81].

Exacerbations of COPD accelerate lung function decline and are a leading cause of hospitalization and increased mortality risk [82]. Viral respiratory infections are a known trigger of COPD exacerbations and account for 40–60% of the exacerbations of infectious etiology [82]. A few retrospective studies have documented the clinical burden of influenza in subjects with chronic lung diseases [82–85]. According to data from the Canadian Immunization Research Network Serious Outcomes Surveillance, laboratory-confirmed influenza was identified in 38.5% of COPD patients during the period 2011–2015, and those who were positive for influenza had higher rates of crude mortality (9.7 vs 7.9%; *p* = 0.047) and critical illness (17.2 vs 12.1%; *p* < 0.001) compared with COPD patients without influenza [82].

#### Influenza vaccination in chronic respiratory diseases

Although data on the effectiveness of influenza vaccines in patients with asthma are limited, a few studies have demonstrated protective effects against influenza and influenza-related hospitalization, and a reduction of asthma attacks after vaccination [81]. In the population study from Canada, influenza-related hospitalization was reduced by 37.5% in vaccinated COPD patients compared with unvaccinated subjects [82]. The effectiveness of influenza vaccination in reducing influenza-related adverse outcomes in patients with chronic lung disease is also documented in a small double-blind RCT from Thailand [86], as well as cohort studies [12, 87].

### Chronic kidney disease

Despite limited evidence about the impact of influenza on subjects with non-respiratory chronic diseases, such as kidney or liver disease, available data indicate that these populations should be considered at high risk for influenza-related complications and, consequently, should receive yearly influenza vaccinations, as recommended by public health authorities [5]. Patients with end-stage renal disease (ESRD) have impaired functions of both innate and adaptive immune system, with defects involving B- and T-cell function as well as complement activation. Uremia, volume overload, malnutrition, iron accumulation and comorbidities are other factors contributing to immune dysfunction, in association with systemic inflammation and oxidative stress, ultimately lowering host defenses and predisposing ESRD patients to a higher incidence and more severe course of infectious diseases [88, 89]. Mortality associated with pulmonary infection was reported to be tenfold higher in ESRD patients compared with the general population [88]. Increased hospitalization and mortality rates were found in dialysis patients, compared with healthy subjects, during the A(H1N1) pandemic [89]. A study based on the US CDC ILI Surveillance Network and the Medicare/Medicaid ESRD database found an association between ILI activity in the community and seasonal variation in all-cause mortality in ESRD patients during the period 2000–2013, with an average number of approximately 1100 deaths per year potentially attributable to ILI [90].

#### Influenza vaccination in chronic kidney disease

Overall, immunogenicity studies seem to suggest that the immune response to influenza vaccines is lower in ESRD patients compared with healthy subjects [88]. A few studies indicate positive outcomes of vaccination in patients with chronic kidney disease, especially regarding all-cause or CV mortality, hospitalization and ICU admission [91]. According to data from the US Renal Data System relative to the 1997–1999 period, influenza vaccination reduced mortality risk in both peritoneal dialysis and hemodialysis patients and decreased hospitalizations in hemodialysis patients compared with unvaccinated subjects [92]. The use of adjuvanted and high-dose vaccines has been suggested to improve immune response in ESRD patients, although studies of the high-dose influenza vaccine in patients undergoing dialysis have shown so far conflicting results [93, 94]. According to Italian surveillance data regarding subjects aged 18–64 years, only 34.2% of patients with renal insufficiency were vaccinated against influenza during the 2020–2021 season [80].

### Chronic liver diseases

The progression of liver disease is characterized by immune dysregulation, and influenza infection has been associated with an increased risk of decompensation in patients with cirrhosis, due to either direct hepatic damage by the virus or immune-mediated damage during systemic infection [95, 96]. In patients with liver disease, influenza infection was associated with a twofold increased risk of hospitalization, compared with healthy subjects, during the 2013–2014 season, and a fivefold increased risk of hospitalization and 17-fold increased risk of mortality during the 2009 pandemic [95]. A case–control study that analyzed data from patients with liver cirrhosis who were hospitalized for respiratory complications during the 2009 pandemic found a higher mortality rate in patients with confirmed A(H1N1) influenza compared with control subjects who were negative (81.8% vs 40%) [96].

#### Influenza vaccination in chronic liver diseases

In patients with liver disease, a meta-analysis of 12 studies (albeit considered of very low quality) found a serological response to influenza vaccination and a 27% reduced risk of hospitalization in vaccinated patients compared with unvaccinated subjects, whereas no significant effect of vaccination was observed on mortality [95]. Only 18.3% of patients with liver diseases, among those aged 18–64 years, were vaccinated against influenza during the 2020–2021 season, according to Italian surveillance data [80]. While there is clearly a need for more studies—and of better quality—to evaluate the protective effects of influenza vaccination in patients with chronic liver diseases, vaccination is highly recommended in this population.

### Vaccines for elderly and high-risk subjects

The document from the Italian Health Ministry that contains recommendations regarding the prevention and control of influenza for the 2023–2024 season identifies the high-dose inactivated influenza vaccine (HD-IIV) and the MF59^®^-adjuvanted inactivated influenza vaccine (adj-IIV) as the two recommended options for the population aged ≥ 65 years [5]. HD-IIV is a quadrivalent split-virus vaccine containing two type A strains (H1N1 and H3N2) and two type B strains. It contains 60 μg hemagglutinin per strain (a fourfold increased amount of hemagglutinin per strain compared with standard-dose inactivated influenza vaccines [SD-IIV]) to ensure a greater immune response and therefore increased efficacy. Adj-IIV is a quadrivalent vaccine containing MF59^®^ as adjuvant, an oil-in-water emulsion of squalene oil. The adjuvant is designed to promote an adequate immune response while using a reduced amount of antigen.

Several immunogenicity studies have demonstrated the greater immunogenicity of HD-IIV compared with SD-IIV in adults aged ≥ 60 years [97]. HD-IIV was first approved (as a trivalent formulation) in 2009 in the US and is the only influenza vaccine globally licensed for use in the elderly population to have demonstrated greater efficacy in preventing laboratory-confirmed influenza, compared with SD-IIV, in an RCT [98].

### Evaluation of influenza vaccines with GRADE methodology

Numerous health authorities have performed systematic reviews for assessment of the efficacy, effectiveness and safety of currently available vaccines (Table 4) [28, 99–103]. In particular, newer and enhanced vaccines (comprising HD-IIV, adj-IIV, cell culture-based IIV and recombinant influenza vaccine) were reviewed in a technical report by the ECDC, which included RCTs and non-randomized studies of interventions (excluding studies conducted during pandemic seasons) published up to February 2020 [100]. The main efficacy or effectiveness outcomes were laboratory-confirmed influenza cases, mortality or hospitalization related to laboratory-confirmed influenza, and CVD or pneumonia/lower respiratory tract disease associated with laboratory-confirmed influenza. Notably, the Grade of Recommendations, Assessment, Development and Evaluation (GRADE) system was used to evaluate the certainty of evidence for each of the main outcomes considered [104]. The main findings of the ECDC report relative to HD-IIV and adj-IIV (the only vaccines that are specifically indicated for the elderly population) are summarized below:

**Table 4:** Evaluation of the scientific evidence* of the efficacy of different types of vaccines against laboratory-confirmed influenza in the elderly by international immunization advisory boards (Modified from Redondo et al. [99])

Advisory board (country-publication date)	HD-IIV	Adj-IIV	cc-IIV	RIV
NACI (Canada-2018) [28]	A (Maximum)	I (Insufficient)^#^	I (Insufficient)	B (Limited)
ECDC (European Union/European Economic Area-2020) [100]	Moderate certainty	No evidence	No evidence	Moderate certainty
STIKO (Germany-2021) [101]	High certainty	Low certainty	Low certainty	Moderate certainty
ATAGI-NCIRS (Australia-2020–2022) [102]	Moderate certainty low certainty^§^	Very low certainty	Very low certainty^§§^	Not evaluated
ACIP (USA-2022) [103]	High certainty	Moderate certainty^#^	Not evaluated	Moderate certainty

*GRADE-based assessments except for Canada. NACI followed a methodology similar to GRADE, with ratings of A (maximum certainty), B (limited certainty) and I (insufficient certainty)

^#^Not specified if laboratory confirmed

^§^Patient record documenting laboratory-confirmed influenza

^§§^Evaluated in people aged ≥ 18 years

*ACIP* Advisory Committee on Immunization Practices; *Adj-IIV* adjuvanted inactivated influenza vaccine; *ATAGI-NCIRS* Australian Technical Advisory Group on Immunisation-National Centre For Immunisation Research And Surveillance; *cc-IIV* cell culture-based inactivated influenza vaccine; *ECDC* European Centre for Disease Prevention and Control; *HD-IIV* high-dose inactivated influenza vaccine; *NACI* National Advisory Committee on Immunization; *RIV* recombinant inactivated influenza vaccine; *STIKO* Standing Committee on Vaccination

*HD-IIV:* 36 studies were included in the review. Trivalent HD-IIV was found to have higher efficacy in preventing influenza compared with trivalent SD-IIV in subjects aged ≥ 65 years (relative vaccine efficacy 24.2%, 95% CI 9.7–36.5%). The evidence, which was considered of moderate certainty, was based on one study, a post-licensure phase 3b-4 double-blind RCT that enrolled community-dwelling participants (*n* = 31,989) from 126 centers in the USA and Canada who received either trivalent HD-IIV or trivalent SD-IIV over two influenza seasons (2011–2012 and 2012–2013) [98]. Notably, 67% of participants had at least one chronic coexisting disease (mainly coronary artery disease). Higher efficacy of HD-IIV against respiratory illness, all-cause hospitalization, serious cardiorespiratory events and pneumonia was also demonstrated [105]. Data about additional outcomes were provided by a single-blind, pragmatic, cluster-RCT in nursing-home residents during the 2013–2014 season [106], and by cohort studies [100]. In the RCT, Gravenstein et al. found a higher vaccine efficacy for trivalent HD-IIV compared with trivalent SD-IIV against respiratory-related hospitalization (relative vaccine efficacy 12.7%, 95% CI 1.8–22.4%) and pneumonia-related hospitalization (20.9%, 95% CI 4.7–73.3%) [106]. The cohort studies document an overall larger effect with HD-IIV versus SD-IIV for influenza-related hospitalizations, influenza- or pneumonia-related hospitalizations, influenza-related hospital encounters and influenza-related office visits (low-certainty evidence for all outcomes) [100]. Pooled estimates of safety data show that trivalent and quadrivalent HD-IIV were associated with significantly higher rates of local and systemic adverse events compared with trivalent and quadrivalent SD-IIV: combined local reactions (risk ratio [RR] 1.40), injection-site pain (RR 1.56), swelling (RR 2.20), induration (RR 1.63), headache (RR 1.35), chills (RR 1.73) and malaise (RR 1.28) [100].

*Adj-IIV:* 48 studies provided suitable data for the ECDC review. No efficacy data were identified that reported results relating to adj-IIV versus any comparator (other vaccines, placebo or no vaccination). As for relative vaccine effectiveness, no significant difference was found in the limited number of studies that compared adj-IIV with other vaccines, suggesting a lack of evidence of increased benefits over non-adjuvanted vaccines in preventing influenza. Adj-IIV was found to be significantly more effective in the prevention of laboratory-confirmed influenza compared with no vaccination (VE 44.9%, 95% CI 22.7–60.8%), except against influenza A(H3N2) (VE 10.6%, 95% CI − 24.5–35.7%), with low- or very low-certainty evidence [100]. As for additional outcomes (from the results of matched case–control and cohort studies), adj-IIV appeared to be superior to no vaccination against influenza-related hospitalization, influenza- or pneumonia-related hospitalization and against ILI. Limited data suggested that adj-IIV may be more effective, compared with non-adjuvanted vaccines, in reducing the risk of influenza- or pneumonia-related hospitalization, influenza-related hospital encounters and ILI [100]. According to pooled safety data, trivalent adj-IIV was associated with a greater number of combined local adverse events compared with trivalent non-adjuvanted vaccines (RR 1.90), injection-site pain (RR 2.02), combined systemic reactions (RR 1.18), myalgia (RR 1.71), fever (RR 1.97) and chills (RR 1.70) [100].

In conclusion, the ECDC report highlighted an overall limited evidence base for the efficacy and effectiveness of newer influenza vaccines. Regarding adj-IIV, there was an absence of high-quality evidence of their efficacy. Collective data for efficacy and effectiveness of HD-IIV suggested that they might provide better protection compared with SD-IIV or no vaccination against laboratory-confirmed influenza or other related outcomes, although caution is needed when interpreting study results. Both adj-IIV and HD-IIV appeared to be well tolerated, despite a higher frequency of solicited local and systemic reactions compared with SD-IIV.

### Enhanced influenza vaccines: what evidence says

Available literature data do not provide conclusive evidence about the relative effectiveness of HD-IIV and adj-IIV. There are no RCTs comparing HD-IIV and adj-IIV, and the results of the few studies that have compared the two vaccines show conflicting results [107]. Health authorities, therefore, have analyzed the evidence supporting the effectiveness of the two types of vaccine. The efficacy of vaccination with HD-IIV against laboratory-confirmed influenza in elderly subjects is supported by a higher quality of evidence compared with adj-IIV vaccination, as documented in the assessments of all the main health authorities. The clinical evaluation of HD-IIV is based on assessment of relevant outcomes (prevention of laboratory-confirmed influenza and severe complications) and a robust methodology, with RCTs in clinical and real-world settings [98, 106, 108]. Numerous cohort studies confirm the superior benefits of HD-IIV relative to SD-IIV over most influenza seasons and across different populations (Table 5) [98, 106, 108–113]. In a meta-analysis of 21 randomized and observational studies that provided data about 12 influenza seasons (from 2009 to 2022) in over 45 million subjects aged ≥ 65 years, HD-IIV was found to be more effective than SD-IIV in protecting against ILI (relative VE [rVE] 14.3%), influenza-related hospitalization (rVE 11.2%), respiratory-related hospitalization (rVE 14.7%), CV-related hospitalization (rVE 12.8%), pneumonia-related hospitalization (27.8%) and all-cause hospitalization (rVE 8.2%). Furthermore, HD-IIV was found to be more effective than SD-IIV in reducing influenza and associated outcomes irrespective of age and circulating influenza strains [114]. The results of a recent pragmatic, randomized feasibility trial, which used an innovative study design (with randomization being integrated into a real-life vaccination practice and data collected using a national health registry) also indicate a lower incidence of hospitalizations and mortality in subjects receiving quadrivalent HD-IIV versus quadrivalent SD-IIV [108].

**Table 5:** Studies investigating the efficacy/effectiveness of high-dose vaccines versus other vaccines in preventing influenza or influenza-related adverse outcomes

Authors [name] country-period	Study design (blinding)	Participants [age-% female]	Intervention	Main findings
DiazGranados et al. [98] USA–Canada (126 centers)-2 seasons (2011–2013)	RCT (double-blind)	31,989 [≥ 65 yrs, mean 73.3–57.1%/56%]	Trivalent HD-IIV versus trivalent SD-IIV	HD-IIV was significantly more efficacious than SD-IIV against laboratory-confirmed influenza (vaccine efficacy 24.2% [95% CI 9.7–36.5%])
Gravenstein et al. [106] USA (823 nursing homes)-1 season (2013–2014)	Cluster-RCT (single-blind)	53,008 residents of nursing homes (38,256 with Medicare data) [≥ 65 yrs, mean 83.6–72%]	Trivalent HD-IIV versus trivalent SD-IIV	The incidence of respiratory-related hospitalizations was significantly lower in nursing homes where residents received HD-IIV versus SD-IIV (RR 0.873 [95% CI 0.776–0.982; *p* = 0.023], as was the incidence of pneumonia-related hospitalizations (RR 0.791 [95% CI 0.267–0.953; *p* = 0.013])
Johansen et al. [DANFLU-1] [108] Denmark-1 season (2021–2022)	Pragmatic RCT (open) based on Danish Health Data registries	12,477 [65–79 yrs, mean 71.7–47.1%]	Quadrivalent HD-IIV versus quadrivalent SD-IIV	HD-IIV was more effective than SD-IIV in preventing influenza- or pneumonia-related hospitalizations (rVE 64.4% [95% CI 24.4–84.6%]) and all-cause mortality (rVE 48.9% [95% CI 11.5–71.3%])
Shay et al. [109] USA-2 seasons (2012–2014)	Retrospective, cohort study based on Medicare data	5,797,090 participants [≥ 65 yrs- 57.9–59.7%]	Trivalent HD-IIV versus trivalent SD-IIV	HD-IIV was significantly more effective than SD-IIV in preventing postinfluenza deaths in 2012–2013 (rVE 36.4% [95% CI 9–55.6%]) but not in 2013–14 (rVE 2.5% [95% CI − 46.8–35.3%])
Lu et al. [110] USA-6 seasons (2012–2018)	Retrospective, cohort study based on Medicare data	13,770,207 participants receiving HD-IIV and 6,151,913 receiving SD-IIV [≥ 65 yrs-58.3%/59.7%]	Trivalent HD-IIV versus trivalent or quadrivalent SD-IIV	HD-IIV was significantly more effective than SD-IIV in preventing influenza-related hospital encounters in 2012–13 (rVE 23.1% [95% CI 17.6–28.3%]), 2013–14 (rVE 15.3% [ 95% CI 7.8–22.3%]), 2014–15 (rVE 8.9% [95% CI 5.6–12.1%]) and 2016–17 (rVE 12.6% [95% CI 6.3–18.4%]), and at least as effective as SD-IIV in the other 2 seasons. HD-IIV was more effective than SD-IIV in subjects aged ≥ 85 yrs across all seasons
Izurieta et al. [111] USA-1 season (2017–2018)	Retrospective, cohort study based on Medicare data	Approximately 13 million participants (trivalent HD-IIV recipients: 63%; quadrivalent SD-IIV: 14%; trivalent adj-IIV:11%; trivalent SD-IIV: 7%; quadrivalent cc-IIV: 5% [≥ 65 yrs- 57.9–59.2%]	Trivalent HD-IIV versus trivalent or quadrivalent SD-IIV versus trivalent adj-IIV versus quadrivalent cc-IIV	There was a low overall vaccine effectiveness. Cc-IIV was more effective than egg-based quadrivalent vaccines in preventing influenza-related hospital encounters (rVE 10% [95% CI 7–13%]). In a 5-way comparison, cc-IIV and HD-IIV were significantly more effective than SD-IIV and adj-IIV in preventing influenza-related hospital encounters. The rVE estimates for HD-IIV were 9% (95% CI 7.2–10.6%) versus quadrivalent SD-IIV, 8.7% (95% CI 6.5–10.9%) versus trivalent SD-IIV and 5.3% (95% CI 3.3–7.3%) versus adj-IIV
Young-Xu et al. [112] USA-3 seasons (2012–2015)	Retrospective, cohort study based on Veterans Health Administration and Medicare data	569,552 participants (HD-IIV recipients: 36%, SD-IIV: 64%) [≥ 65 yrs-1%]	Trivalent HD-IIV versus SD-IIV	During high influenza periods, vaccination with HD-IIV was associated with a lower risk of influenza/pneumonia-related death (pooled rVE 36% [95% CI 10–62%]) and cardiorespiratory death (pooled rVE 25% [95% CI 12–38%]) compared with vaccination with SD-IIV
Van Aalst et al. [113] USA-2 seasons (2016–2018)	Retrospective, cohort study	Members of a national managed care organization, 1,900,920 receiving HD-IIV and 223,793 receiving adj-IIV [≥ 65 yrs-58%]	Trivalent HD-IIV versus trivalent adj-IIV	Vaccination with HD-IIV was associated with lower rates of hospitalization due to respiratory disease (pooled rVE 12% [95% CI 3.3–20%]) or cardiorespiratory disease (pooled rVE 7.0% [95% CI 2.3–12%]) compared with adj-IIV

*Adj-IIV* MF59-adjuvanted inactivated influenza vaccine; *cc-IIV* cell culture-based inactivated influenza vaccine; *CI* confidence interval; *DANFLU-1* feasibility of randomizing Danish citizens aged 65–79 years to high-dose quadrivalent influenza vaccine versus standard-dose quadrivalent influenza vaccine in a pragmatic registry-based setting; *HD-IIV* high-dose inactivated influenza vaccine; *HF* heart failure; *MI* myocardial infarction; *RCT* randomized controlled trial; *RR* relative risk; *rVE* relative vaccine effectiveness; *SD-IIV* standard-dose inactivated influenza vaccine

Taken together, literature findings suggest that HD-IIV is consistently more effective than SD-IIV in the prevention of influenza and influenza-related complications irrespective of circulating strains, in both controlled and real-world settings. Only a limited number of studies, however, have specifically investigated comparative vaccine efficacy or effectiveness in high-risk populations other than elderly subjects.

The robust evidence supporting the superior efficacy of HD-IIV compared with SD-IIV in subjects aged ≥ 65 years makes HD-IIV a preferable option in the elderly population. Quadrivalent HD-IIV is included among the recommended options for elderly people in most countries, and considered the preferred option by the German Standing Vaccination Committee. It should be noted that many factors beyond proven efficacy and tolerability are taken into account for proposals of vaccination schemes, such as accessibility and cost considerations. For these reasons, most national recommendations include both HD-IIV and adj-IIV.

## Conclusions

A large body of evidence documents the benefits of vaccination in reducing severe illness and complications associated with influenza infection in elderly subjects and individuals of any age in high-risk groups, in particular those with CVD, diabetes and chronic lung diseases. Notwithstanding the importance of an accurate prediction of circulating viral strains and the degree of antigenic drift as key determinants of vaccine effectiveness, different vaccine types have been associated with varying levels of protection. HD-IIV have been specifically developed to overcome the problems associated with immunosenescence in elderly subjects. The benefits of HD-IIV in this vulnerable population have been demonstrated in RCTs and confirmed in observational studies that have explored the protective effects of this vaccine against relevant clinical outcomes across different seasons and settings. Vaccination should be considered as an integral part of prevention strategies in subjects with CVD, diabetes or other chronic diseases, but surveillance data show that immunization rates remain suboptimal in high-risk individuals. All clinicians, both general practitioners and specialists, should take a more proactive role in increasing vaccine confidence and adherence among their patients.

### Supplementary Information

Below is the link to the electronic supplementary material.

Supplementary file1 (DOCX 28 KB)

## Abbreviations

adj-IIV: MF59^®^-adjuvanted inactivated influenza vaccine
AMI: Acute myocardial infarction
ARI: Acute respiratory illness
BMI: Body mass index
cc-IIV: Cell culture-based inactivated influenza vaccine
CDC: Centers for disease control and prevention
CI: Confidence interval
CV: Cardiovascular
CVD: Cardiovascular disease
COPD: Chronic obstructive pulmonary disease
DALY: Disability-adjusted life years
ECDC: European Centre for Disease Control and Prevention
ESRD: End-stage renal disease
GRADE: Grade of Recommendations, Assessment, Development and Evaluation
HA: Hemagglutinin
HR: Hazard ratio
HD-IIV: High-dose inactivated influenza vaccine
HF: Heart failure
IAMI: Influenza vaccination after myocardial infarction study
ICU: Intensive care unit
IHD: Ischemic heart disease
ILI: Influenza-like illness
IR: Incidence ratio
IVVE: Influenza vaccine in patients with heart failure to reduce adverse cardiovascular events study
MACE: Major adverse cardiovascular events
MI: Myocardial infarction
PARADIGM-HF: Prospective comparison of ARNI with ACEI to determine impact on global mortality and morbidity in heart failure study
OR: Odds ratio
RCT: Randomized controlled trial
RIV: Recombinant influenza vaccine
RR: Relative risk/risk ratio
RTI: Respiratory tract infection
SCA: Sudden cardiac arrest
SD-IIV: Standard-dose inactivated influenza vaccine
VE/rVE: Vaccine effectiveness/relative vaccine effectiveness
WHO: World Health Organization
