# Supplementary material for: Influenza vaccination for elderly, vulnerable and high-risk subjects: a narrative review and expert opinion
Source: Intern Emerg Med. 2023 Oct 27;19(3):619–40. doi: 10.1007/s11739-023-03456-9 (PMC11039544; doi:10.1007/s11739-023-03456-9)
Supplement: Supplementary file 1 — Supplementary file1 (DOCX 28 KB) [file 11739_2023_3456_MOESM1_ESM.docx]

## Supplementary material

## Box

Influenza vaccination for elderly, vulnerable and high-risk subjects: a narrative review and expert opinion

Raffaele Antonelli Incalzi, Agostino Consoli, Pierluigi Lopalco, Stefania Maggi, Giorgio Sesti, Nicola Veronese, Massimo Volpe

**Box** - Persons at high risk for influenza-related complications or hospitalization (Italian Ministry of Health)^5^

| - Women who are pregnant or post-partum at the start of the epidemic season - Subjects 6 months-65 years of age with health conditions that increase the risk of influenza-related complications:  1. chronic respiratory diseases (including severe asthma, bronchopulmonary dysplasia, cystic fibrosis and chronic obstructive pulmonary disease) 2. cardiovascular diseases, including congenital or acquired cardiac diseases 3. diabetes mellitus and other metabolic diseases (including obese subjects with BMI>30) 4. chronic renal/adrenal insufficiency 5. hematopoietic organ diseases or hemoglobinopathies 6. cancer and subjects undergoing chemotherapy 7. congenital or acquired immune compromising diseases, drug- or HIV-induced immune suppression 8. chronic inflammatory diseases and intestinal malabsorption syndromes 9. planned major surgical interventions 10. diseases posing an increased risk of aspiration pneumonia (e.g. neuromuscular diseases) 11. chronic liver disease  - Subjects aged ≥65 years - Children and adolescents undergoing long-term treatment with acetyl salicylic acid, at risk of influenza-related Reye syndrome - Residents of long-term care facilities of any age - Family members and contacts (adults and children) of subjects at high risk of complications (irrespective of vaccination status of high-risk subject)   *BMI* body mass index, *HIV* human immunodeficiency virus |
| --- |

# Supplementary information—Further Reading

Influenza vaccination for elderly, vulnerable and high-risk subjects: a narrative review and expert opinion

Raffaele Antonelli Incalzi, Agostino Consoli, Pierluigi Lopalco, Stefania Maggi, Giorgio Sesti, Nicola Veronese, Massimo Volpe

# Introduction

Beck CR, McKenzie BC, Hashim AB, Harris RC; University of Nottingham Influenza and the ImmunoCompromised (UNIIC) Study Group, Nguyen-Van-Tam JS (2012) Influenza vaccination for immunocompromised patients: systematic review and meta-analysis by etiology. J Infect Dis 206(8):1250-1259. <https://doi.org/10.1093/infdis/jis487>

European Medicines Agency. Influenza vaccines – non-clinical and clinical module. 2016. Available from: <https://www.ema.europa.eu/en/influenza-vaccines-non-clinical-clinical-module-scientific-guideline> [Accessed 25 Jan 23].

Hayward AC, Fragaszy EB, Bermingham A, et al; Flu Watch Group (2014) Comparative community burden and severity of seasonal and pandemic influenza: results of the Flu Watch cohort study. Lancet Respir Med 2(6):445-54. <https://doi.org/10.1016/s2213-2600(14)70034-7>

Lai PL, Panatto D, Ansaldi F, et al. Burden of the 1999-2008 seasonal influenza epidemics in Italy:comparison with the H1N1v (A/California/07/09) pandemic (2011) Hum Vaccin 7(Suppl):217-225. <https://doi.org/10.4161/hv.7.0.14607>

Osterholm MT, Kelley NS, Sommer A, Belongia EA (2012) Efficacy and effectiveness of influenza vaccines: a systematic review and meta-analysis. Lancet Infect Dis 12(1):36-44. <https://doi.org/10.1016/s1473-3099(11)70295-x>

Ting EEK, Sander B, Ungar WJ (2017) Systematic review of the cost-effectiveness of influenza immunization programs. Vaccine 35(15):1828-1843. <https://doi.org/10.1016/j.vaccine.2017.02.044>

**Impact of influenza on high-risk populations and benefits of vaccination**

Altay M, Ateş İ, Altay FA, Kaplan M, Akça Ö, Özkara A (2016) Does education effect the rates of prophylactic vaccination in elderly diabetics? Diabetes Res Clin Pract 120:117-123. <https://doi.org/10.1016/j.diabres.2016.08.002>

Barker WH, Borisute H, Cox C (1998) A study of the impact of influenza on the functional status of frail older people. Arch Intern Med 158(6):645-50. <https://doi.org/10.1001/archinte.158.6.645>

Barnes M, Heywood AE, Mahimbo A, Rahman B, Newall AT, Macintyre CR (2015) Acute myocardial infarction and influenza: a meta-analysis of case-control studies. Heart 101(21):1738-47. <https://doi.org/10.1136/heartjnl-2015-307691>

Carey IM, Critchley JA, DeWilde S, Harris T, Hosking FJ, Cook DG (2018) Risk of Infection in Type 1 and Type 2 Diabetes Compared With the General Population: A Matched Cohort Study. Diabetes Care 41(3):513-21. <https://doi.org/10.2337/dc17-2131>

Fabiani M, Volpe E, Faraone M, Bella A, Pezzotti P, Chini F (2020) Effectiveness of influenza vaccine in reducing influenza-associated hospitalizations and deaths among the elderly population; Lazio region, Italy, season 2016-2017. Expert Rev Vaccines 19(5):479-489. <https://doi.org/10.1080/14760584.2020.1750380>

Fagnoul D, Pasquier P, Bodson L, Ortiz JA, Vincent JL, De Backer D (2013) Myocardial dysfunction during H1N1 influenza infection. J Crit Care 28(4):321-7. <https://doi.org/10.1016/j.jcrc.2013.01.010>

Frasca D, Diaz A, Romero M, et al (2013) Young and elderly patients with type 2 diabetes have optimal B cell responses to the seasonal influenza vaccine. Vaccine 31(35):3603-10. <https://doi.org/10.1016/j.vaccine.2013.05.003>

Gozalo PL, Pop-Vicas A, Feng Z, Gravenstein S, Mor V (2012) Effect of influenza on functional decline. J Am Geriatr Soc 60(7):1260-7. <https://doi.org/10.1111/j.1532-5415.2012.04048.x>

Jiménez-García R, Hernández-Barrera V, Rodríguez-Rieiro C, et al (2013) Hospitalizations from pandemic Influenza [A(H1N1)pdm09] infections among type 1and 2 diabetes patients in Spain. Influenza Other Respir Viruses 7(3):439-447. <https://doi.org/10.1111/j.1750-2659.2012.00419.x>

Lees C, Godin J, McElhaney JE, et al (2020) Frailty Hinders Recovery From Influenza and Acute Respiratory Illness in Older Adults. J Infect Dis 222(3):428-437. <https://doi.org/10.1093/infdis/jiaa092>

Macintyre CR, Heywood AE, Kovoor P, et al (2013) Ischaemic heart disease, influenza and influenza vaccination: a prospective case control study. Heart 99(24):1843-8. <https://doi.org/10.1136/heartjnl-2013-304320>

Neidich SD, Green WD, Rebeles J, et al (2017) Increased risk of influenza among vaccinated adults who are obese. Int J Obes (Lond) 41(9):1324-1330. <https://doi.org/10.1038/ijo.2017.131>

Samson SI, Konty K, Lee WN, et al (2021) Quantifying the Impact of Influenza Among Persons With Type 2 Diabetes Mellitus: A New Approach to Determine Medical and Physical Activity Impact. J Diabetes Sci Technol 15(1):44-52. <https://doi.org/10.1177/1932296819883340>

Seo YB, Baek JH, Lee J, et al (2015) Long-Term Immunogenicity and Safety of a Conventional Influenza Vaccine in Patients with Type 2 Diabetes. Clin Vaccine Immunol 22(11):1160-5. <https://doi.org/10.1128/cvi.00288-15>

Sheridan PA, Paich HA, Handy J, et al (2015) The antibody response to influenza vaccination is not impaired in type 2 diabetics. Vaccine 33(29):3306-13. <https://doi.org/10.1016/j.vaccine.2015.05.043>

Verger P, Cortaredona S, Pulcini C, Casanova L, Peretti-Watel P, Launay O (2015) Characteristics of patients and physicians correlated with regular influenza vaccination in patients treated for type 2 diabetes: a follow-up study from 2008 to 2011 in southeastern France. Clin Microbiol Infect 21(10):930.e1-9. <https://doi.org/10.1016/j.cmi.2015.06.017>

Wellen KE, Hotamisligil GS (2005) Inflammation, stress, and diabetes. J Clin Invest 115(5):1111-1119. <https://doi.org/10.1172/jci25102>

Zhao X, Gang X, He G, et al (2020) Obesity Increases the Severity and Mortality of Influenza and COVID-19: A Systematic Review and Meta- Analysis. Front Endocrinol (Lausanne) 11:595109. <https://doi.org/10.3389/fendo.2020.595109>

Zwaans WA, Mallia P, van Winden ME, Rohde GG (2014) The relevance of respiratory viral infections in the exacerbations of chronic obstructive pulmonary disease—a systematic review. J Clin Virol 61(2):181-188. <https://doi.org/10.1016/j.jcv.2014.06.025>

**Vaccines for elderly and high-risk subjects**

Balasubramani GK, Choi WS, Nowalk MP, et al; US Flu VE Network Investigators (2020) Relative effectiveness of high dose versus standard dose influenza vaccines in older adult outpatients over four seasons, 2015-16 to 2018-19. Vaccine 38(42):6562-6569. <https://doi.org/10.1016/j.vaccine.2020.08.011>

Comber L, O Murchu E, Jordan K, et al (2023) Systematic review of the efficacy, effectiveness and safety of high-dose seasonal influenza vaccines for the prevention of laboratory-confirmed influenza in individuals ≥18 years of age. Rev Med Virol 33(3):e2330. <https://doi.org/10.1002/rmv.2330>

Diaco M, Chang LJ, Seet B, et al (2021) Introductory paper: High-dose influenza vaccine. Vaccine;39 (Suppl 1):A1-A5. <https://doi.org/10.1016/j.vaccine.2020.09.005>

DiazGranados CA, Dunning AJ, Robertson CA, Talbot HK, Landolfi V, Greenberg DP (2015) Efficacy and immunogenicity of high-dose influenza vaccine in older adults by age, comorbidities, and frailty. Vaccine 33(36):4565-4571. <https://doi.org/10.1016/j.vaccine.2015.07.003>

DiazGranados CA, Robertson CA, Talbot HK, Landolfi V, Dunning AJ, Greenberg DP (2015) Prevention of serious events in adults 65 years of age or older: A comparison between high-dose and standard-dose inactivated influenza vaccines. Vaccine 33(38):4988-93. <https://doi.org/10.1016/j.vaccine.2015.07.006>

Domnich A, de Waure C (2022) Comparative effectiveness of adjuvanted versus high-dose seasonal influenza vaccines for older adults: a systematic review and meta-analysis. Int J Infect Dis 122:855-63. <https://doi.org/10.1016/j.ijid.2022.07.048>

Doyle JD, Beacham L, Martin ET, et al (2021) Relative and Absolute Effectiveness of High-Dose and Standard-Dose Influenza Vaccine Against Influenza-Related Hospitalization Among Older Adults-United States, 2015-2017. Clin Infect Dis 72(6):995-1003. <https://doi.org/10.1093/cid/ciaa160>

Goldet G, Howick J (2013) Understanding GRADE: an introduction. J Evid Based Med 6(1):50-54. <https://doi.org/10.1111/jebm.12018>

Izurieta HS, Thadani N, Shay DK, et al (2015) Comparative effectiveness of high-dose versus standard-dose influenza vaccines in US residents aged 65 years and older from 2012 to 2013 using Medicare data: a retrospective cohort analysis. Lancet Infect Dis 15(3):293-300. <https://doi.org/10.1016/s1473-3099(14)71087-4>

Izurieta HS, Chillarige Y, Kelman J, et al (2020) Relative Effectiveness of Influenza Vaccines Among the United States Elderly, 2018-2019. J Infect Dis 222(2):278-287. <https://doi.org/10.1093/infdis/jiaa080>

Izurieta HS, Lu M, Kelman J, et al (2021) Comparative Effectiveness of Influenza Vaccines Among US Medicare Beneficiaries Ages 65 Years and Older During the 2019-2020 Season. Clin Infect Dis 73(11):e4251-e4259. <https://doi.org/10.1093/cid/ciaa1727>

Layton JB, McGrath LJ, Sahrmann JM, et al (2020) Comparative safety of high-dose versus standard-dose influenza vaccination in patients with end-stage renal disease. Vaccine 38(33):5178-86. <https://doi.org/10.1016/j.vaccine.2020.06.020>

Loeb N, Andrew MK, Loeb M, et al (2020) Frailty Is Associated With Increased Hemagglutination-Inhibition Titers in a 4-Year Randomized Trial Comparing Standard- and High-Dose Influenza Vaccination. Open Forum Infect Dis 7(5):ofaa148. <https://doi.org/10.1093/ofid/ofaa148>

Machado MAA, Moura CS, Abrahamowicz M, Ward BJ, Pilote L, Bernatsky S (2021) Relative effectiveness of influenza vaccines in elderly persons in the United States, 2012/2013-2017/2018 seasons. NPJ Vaccines 6(1):108. <https://doi.org/10.1038/s41541-021-00373-w>

Ng TWY, Cowling BJ, Gao HZ, Thompson MG (2019) Comparative Immunogenicity of Enhanced Seasonal Influenza Vaccines in Older Adults: A Systematic Review and Meta-analysis. J Infect Dis 219(10):1525-1535. <https://doi.org/10.1093/infdis/jiy720>

O Murchu E, Comber L, Jordan K, et al (2023) Systematic review of the efficacy, effectiveness and safety of MF59^®^ adjuvanted seasonal influenza vaccines for the prevention of laboratory-confirmed influenza in individuals ≥18 years of age. Rev Med Virol 33(3):e2329. <https://doi.org/10.1002/rmv.2329>

Pelton SI, Divino V, Postma MJ, et al (2021) A retrospective cohort study assessing relative effectiveness of adjuvanted versus high-dose trivalent influenza vaccines among older adults in the United States during the 2018-19 influenza season. Vaccine 39(17):2396-407. <https://doi.org/10.1016/j.vaccine.2021.03.054>

Ruiz-Aragón J, Márquez-Peláez S, Gani R, Alvarez P, Guerrero-Luduena R 2022) Cost-Effectiveness and Burden of Disease for Adjuvanted Quadrivalent Influenza Vaccines Compared to High-Dose Quadrivalent Influenza Vaccines in Elderly Patients in Spain. Vaccines (Basel) 10(2):176. <https://doi.org/10.3390/vaccines10020176>

Schmader KE, Liu CK, Harrington T, et al (2021) Safety, Reactogenicity, and Health-Related Quality of Life After Trivalent Adjuvanted vs Trivalent High-Dose Inactivated Influenza Vaccines in Older Adults: A Randomized Clinical Trial*.* JAMA Netw Open 4(1):e2031266. <https://doi.org/10.1001/jamanetworkopen.2020.31266>

Vardeny O, Kim K, Udell JA, et al; INVESTED Committees and Investigators (2021) Effect of High-Dose Trivalent vs Standard-Dose Quadrivalent Influenza Vaccine on Mortality or Cardiopulmonary Hospitalization in Patients With High-risk Cardiovascular Disease: A Randomized Clinical Trial. JAMA 325(1):39-49. <https://doi.org/10.1001/jama.2020.23649>
